# Antimicrobial and molluscicidal activities of Egyptian soil-derived *Streptomyces rochei*

**DOI:** 10.1186/s13568-025-01927-0

**Published:** 2025-08-22

**Authors:** Nora Elfeky, Aya Abd Elsalam, Sabha El-sabbagh, Asmaa Abdel-Motleb

**Affiliations:** 1 Botany and Microbiology Department, Faculty of Science, Menoufia University, Menoufia, Egypt; 2 Environmental Research Department, Theodor Bilharz Research Institute, Giza, Egypt

**Keywords:** Antimicrobial activity, Molluscicidal activity, *Streptomyces rochei*, Scanning electron microscope

## Abstract

Actinomycetes, are abundant sources of bioactive secondary metabolites with potential antibacterial and molluscicidal effects. This work aims to isolate and characterize actinomycetes strains from Egyptian soil, emphasizing the evaluating their antibacterial efficiency, optimizating their metabolite production, and assessing molluscicidal activities against *Biomphalaria alexandrina* (*B. alexandrina*), an intermediate host of *Schistosoma mansoni* (*S. mansoni*). Nineteen actinomycetes isolates were screened for antimicrobial activity against *Escherichia coli (E. coli)*,* Salmonella typhi* (*S. typhi*), *Pseudomonas aeruginosa* (*P. aeruginosa*), *Staphylococcus aureus* (*S. aureus*), *Candida albicans* (*C. albicans*), and *Penicillium marneffei* (*P. marneffei*). Isolate No. 10, later identified as *Streptomyces rochei ASN* (S. *rochei* ) via 16 S rDNA sequencing and phylogenetic analysis, exhibited broad-spectrum antimicrobial activity and was selected for further study. Production media optimization revealed that modified nutritional agar, starch as a carbon source, sodium nitrate as a nitrogen source, and 25 °C incubation maximized antimicrobial metabolite production. Antibacterial activity increased with extended incubation time, peaking at 8 days. Dose-dependent inhibition was observed when different concentrations (20–80%) of cell-free supernatant (CFS) from *S. rochei* ASN were applied to test pathogens, with *S. aureus*,* C. albicans*, and *P. marneffei* being most susceptible. Scanning electron microscopy revealed severe cellular and structural damage in treated microbes. The CFS also demonstrated significant molluscicidal activity, with LC₅₀ and LC₉₀ values of 26.85 ppm and 40.95 ppm, respectively. It inhibited the hatchability of *B. alexandrina* eggs in a concentration- and age-dependent manner, and exerted rapid, dose-dependent mortality against *S. mansoni* miracidia and cercariae. Histological examinations of exposed snails showed degeneration in both hermaphrodite and digestive glands, including disrupted acinar structures, damaged oocytes and spermatocytes, vacuolated digestive cells, and degenerated secretory cells. *S. rochei* ASN, isolated from Egyptian soil, exhibits potent antimicrobial and molluscicidal activities. Its bioactivity is significantly influenced by growth conditions and medium composition. The strain holds promise as a natural biocontrol agent against pathogenic microbes and snail vectors involved in schistosomiasis transmission, warranting further pharmacological and ecological investigations.

**Supplementary Information:**

The online version contains supplementary material available at 10.1186/s13568-025-01927-0.

## Introduction

Antimicrobial resistance (AMR) represents an escalating global health threat, driven primarily by the widespread misuse and overuse of antimicrobial agents, as well as various environmental factors. This has contributed significantly to the emergence and proliferation of resistant bacterial and fungal strains. According to estimates by the World Health Organization (WHO), AMR is currently linked to approximately 700,000 deaths annually. If effective alternative therapeutic strategies are not developed and implemented, this number is projected to rise dramatically, potentially reaching 10 million deaths per year by 2050 (Mancuso et al. 2021; WHO 2023). This alarming trend underscores the urgent need to discover new antimicrobial agents and explore alternative biological control strategies. Among the most promising sources of novel antimicrobials are actinomycetes, particularly members of the *Streptomyces* genus (Elfeky et al. 2025). These Gram-positive filamentous bacteria are renowned for their ability to produce a wide range of bioactive secondary metabolites, including antibiotics, antifungals, and antiparasitic compounds (De Simeis and Serra 2021; Silva et al. 2022). *Streptomyces* spp. accounts for the majority of naturally derived antibiotics used in clinical practice. Their metabolic output is highly influenced by environmental conditions and nutrient availability, especially carbon and nitrogen sources, which can be optimized to enhance metabolite yields (Elfeky et al. 2025: Lertcanawanichakul and Sahabuddeen 2023).

Beyond their antimicrobial activity, actinomycetes have shown promising molluscicidal properties, opening new avenues for controlling parasitic diseases such as schistosomiasis (Xing et al. 2021). This disease continues to affect millions globally, particularly in developing countries, despite ongoing control efforts (WHO 2020). While Egypt has made considerable progress in reducing disease incidence, snail vector control remains a critical component of prevention strategies (WHO 2023). Current reliance on chemical molluscicides raises environmental and safety concerns, prompting the need for biologically derived alternatives (King et al. 2015; Cando et al. 2022). Several studies have demonstrated that actinomycetes possess molluscicidal activity, making them promising candidates for integrated vector management (de Oliveira et al. 2004; Molla et al. 2013; Duval et al. 2015; Paula-Andrade et al. 2019; Ibrahim et al. 2021; Saleh et al. 2022). *B. alexandrina*, a freshwater snail, is the primary intermediate host of *S. mansoni* in Egypt, where its eggs are most prevalent. These eggs are commonly found on aquatic plants in irrigation canals and drains, supporting seasonal increases in snail populations—especially during spring. Disrupting the reproductive cycle—particularly by targeting the eggs—is therefore critical in controlling snail populations and reducing schistosomiasis transmission (Mekawey et al. 2022).

Furthermore, these microorganisms produce enzymes such as chitinases that degrade chitin, a key structural component of fungal cell walls and molluscan exoskeletons, potentially enhancing both antimicrobial and molluscicidal activities (Mathew et al. 2021; Dhole et al. 2021). Despite this potential, few studies have explored the dual bioactivity of actinomycetes in both antimicrobial and vector control applications.

Given the urgent need for novel bioactive agents to combat antimicrobial resistance and control disease vectors, this study was conducted to isolate and characterize actinomycetes from Egyptian agricultural soils and assess their potential as antimicrobial and molluscicidal agents. The research aimed to identify the most biologically active isolate, optimize its culture conditions for enhanced metabolite production, and evaluate its efficacy against a range of human pathogens, *B. alexandrina* snails, their eggs, and the larval stages of *S. mansoni*. Additionally, the study investigated the filtrate extract of isolate’s impact on snail tissue through histopathological analysis and examined the human pathogen structural alterations using scanning electron microscopy (SEM), providing a comprehensive evaluation of its biocontrol capabilities.

## Materials and methods

### Isolation of the actinomycetes

Soil samples were collected from the agricultural region in Ashmun, Menoufia governorate, Egypt. The samples were taken from 15 to 20 cm depth. They were collected in triplicates under sterile conditions according to the standard methods for the examination of soil. Physical treatment was used for the isolation of actinomycetes from soil. Samples were dried at 100 °C for one hour (Meenakshi et al. 2024) and serial dilutions were made from the dried soil samples then 1 ml from each dilution was inoculated on starch agar (soluble starch: 10 g, beef extract: 3 g, agar:12 g, distilled water: 1 L, pH was adjusted to 7.5) (Atlas 2004) and incubated at 28 °C for 7 days. After the incubation period, distinct colonies that exhibited typical actinomycetes morphology (dry, chalky, or powdery colonies with a tough, leathery texture and branching filamentous growth) were picked and repeatedly sub cultured on fresh starch agar plates to ensure purity. A total of 19 purified actinomycete isolates were obtained, and pure cultures were maintained on agar slants at 4 °C for further studies (Abou El-Enain et al. 2023).

### **Source of pathogenic microorganisms**

*Escherichia coli* (ATCC25922), *Pseudomonas aeruginous* (ATCC9027), *Salmonella typhi* (ATCC14028), *Staphylococcus aureus* (ATCC6538), *Candida albicans* (ATCC10231) acquired from the American type culture collection(**ATCC; Rockville**,** MD USA**). *Penicillium marneffei* (RCMB001F022) is a clinical isolate of human pathogenic and was supplied from Al-Azhar University, Egypt, as a pure active culture of agar slant using screw-capped pyrex tubes.

### Screening of antimicrobial effectiveness

The 19 actinomycetes strains were tested for their antimicrobial activity by the agar disc method (Clinical and Laboratory Standards Institute 2020). In this method, the 19 samples were incubated on a nutrient agar medium for 48hs after that, discs were made from the region of unified growth. Nutrient agar plates were inoculated with the pathogenic bacteria and Potato dextrose agar plates were used for the fungi. Actinomycete agar plugs discs (6 mm), taken from evenly streaked plates incubated for 48 h at 28 °C, were placed onto agar plates that had been previously streaked with the test pathogens.The clear zone was observed after the incubation period at 37°C for 24 h for bacteria and *C. albicans* and for 3 days in the case of *P. marneffei*. The isolates with the largest clear zone were chosen for further investigation.

### Molecular identification of the isolated actinomycetes

The identification of the actinomycete isolate (N-10, the highest antimicrobial agent) was confirmed through 16 S rRNA gene sequencing, following the protocol previously described by Gharieb et al. (2024). Briefly, genomic DNA was extracted and purified using the Qiagen PCR Purification Kit (Qiagen Inc., Valencia, CA), and its quality was assessed based on OD260/280 and OD260/230 ratios. PCR amplification of the 16 S rRNA gene was carried out using universal primers 27 F and 1492R, with Dr. MAX Taq DNA Polymerase (Doctor Protein, Korea). The amplified product was purified and sequenced using the Sanger method with BigDye Terminator v3.1 Cycle Sequencing Kit (Applied Biosystems), employing internal primers 785 F and 907R. Sequencing was performed on an ABI PRISM 3730XL Analyzer. The sequencing findings were evaluated via BLAST. Phylogenetic analysis was conducted using MEGA11 software, employing the Maximum Likelihood method for tree construction. Sequence alignment was performed using the ClustalW algorithm integrated within MEGA11. To ensure the reliability of the alignment, positions with less than 95% site coverage—including gaps, missing data, and ambiguous bases—were excluded using the partial deletion option. Evolutionary distances were estimated using the Jukes-Cantor model, and the robustness of the resulting tree was evaluated through bootstrap analysis with 1000 replicates. The final phylogenetic tree, along with the associated bootstrap values, was exported and visualized using the Interactive Tree of Life (iTOL) platform (https://itol.embl.de/) for enhanced graphical presentation and annotation.

The final sequence was submitted to the GenBank database and designated the accession number PQ673648 and was preserved at Assiut University in the Moubasher Mycological Centre, Egypt under strain no AUMC- 695.

### Optimization of culture conditions for assessing antimicrobial activity of *S. rochei*

The optimization of antibacterial efficacy for *S. rochei* was performed utilizing the agar well diffusion assay with cell-free supernatant (CFS). The preparation of seed culture (SC) entailed inoculating five agar discs of the isolate into 100mL modified nutrient broth, followed by incubation at 28 °C for 24 h. A 5 mL aliquot of the SC was inoculated into 100 mL of sterile medium and incubated at 28 °C with agitation (150 rpm) for 7 days. Following incubation, the culture was centrifuged at 6000 rpm for 10 min, and the supernatant was filtered using a 0.45 μm syringe filter to get sterile cell-free supernatant (CFS). For antimicrobial testing, 20 mL of nutrient agar (NA) or potato dextrose agar (PDA) was dispensed into Petri dishes, and 6 mm wells were created. Pathogen strains were seeded onto the plates, and each well was supplemented with 100 µL of cell-free supernatant (CFS). Plates were incubated at 37 °C for 24 h for bacteria, 28 °C for 24 h for *C. albicans*, and 28 °C for 3 days for fungi, following which inhibitory zones were quantified (Vijayakumar et al. 2012).

To optimize antimicrobial activity, various factors were thoroughly assessed using the above-described method to determine the ideal conditions for enhancing the antimicrobial effectiveness of *S. rochei*. The factors included growth media (nutrient agar, starch medium, ISP2, Bennet’s medium), incubation duration (2, 4, 6, and 8 days), carbon sources (glucose, starch, lactose, glycol, and sucrose), nitrogen sources (peptone, casein, urea, NH₄Cl, NaNO₃), and temperature (25 °C, 30 °C, 35 °C) **(**Djinni et al. 2023).

### Impact of different concentrations of *S. rochei* cell-free supernatant on pathogenic microorganisms growth

With minor adjustments for the dry weight assessment, the effect of *S. rochei’s* cell-free filtrate (CFS) on the pathogens’ growth was assessed in accordance with (El-Abyad et al. 1993). Different CFS concentrations (20%, 60%, and 80%) were tested against a fresh-made broth culture of the pathogens. The concentrations of CFS were applied to the pathogens’ fresh broth culture. Then the treated fungus and *C. albicans* were incubated for 72 h and 24 h in potato dextrose broth media, respectively. While the treated bacteria were incubated for 24 h in nutrient broth media (Atlas 2004). Following the incubation period, each test tube was centrifuged for 10 min at 4000 rpm to extract the pellet, which was subsequently dried at 60 °C till reached the fixed weight. Both the fresh and dried weight of the biomass were detected.

### Scanning electron microscope (SEM) analysis of morphological alterations in pathogenic microorganisms treated with *S. rochei* Cell-free supernatant

To investigate the morphological effects of the cell-free supernatant (CFS) of *S. rochei* on *E. coli*, *S. aureus*, and *P. marneffei*, scanning electron microscopy (SEM) analysis was performed. Pathogen liquid cultures were treated with 20% (v/v) CFS and incubated under previously described conditions. Following incubation, cells were harvested by centrifugation at 3000 rpm for 10 min, washed twice with sterilized distilled water, and fixed in 2.5% glutaraldehyde prepared in 0.1 M phosphate-buffered saline (PBS, pH 7.4) at 4 °C for 2 h. Prior to imaging, specimens were sputter-coated with a thin layer of gold to improve conductivity and reduce charging artifacts. SEM imaging was carried out using a JEOL IT200 scanning electron microscope (JEOL, Germany) at the Faculty of Science, Alexandria University, Egypt, with imaging parameters optimized to ensure clear visualization of cellular surface morphology.

### Detection of the molluscicidal activity of *S. rochei*

#### Experimental snails

Adult *B. alexandrina* snails were used in the main experiment. The snails were collected from El-Mansouriya main canal, Giza Governorate, Egypt, (30ο00 N, 31ο10 E) using a hand net, transported to the laboratory and examined for natural trematode infections by exposing them to artificial light at 25 ± 2 °C. Healthy adults were kept in plastic aquaria containing aerated tap water (10 snails per aquarium) for one month before being used in experiments (Boissier and Mone 2000). These snails were fed boiled fresh lettuce leaves and blue-green algae (Nostocmuscarm). The water of the aquaria was renewed two times per week to avoid deterioration, and dead snails were removed daily. The 2nd generation of these snails was used.

#### Molluscicidal effects

A series of concentrations (20, 40, 60, 80 and 100 ppm) that would permit the computation of LC_50_ and LC_90_ values were prepared from SFC of *S. rochei* against *B. alexandrina* (10–12 mm) in three repllicates (10 snails/ 1000 ml) for each concentration (El-Mahdy et al. 2021). A set of control snails was maintained under the same experimental conditions. The exposure period was 24 h followed by another 24 h of recovery at room temperature 25 ± 2 °C. The number of dead snails in each concentration was recorded and the LC_50_, LC_90_, and sub-lethal concentrations were calculated by Probit analysis based on (Abdel-Wareth et al. 2019) using SPSS software (version 20.1, Chicago, IL, USA). The sub-lethal concentration (LC_o_) was calculated as equal to 1/10 LC_50_ (Tantawy 2008).

### Effect of *CFS of S. rochei* on hatchability of *B. alexandrina* eggs

The impact of the tested filtrate on the hatchability of *B. alexandrina* eggs was assessed using three replicates of egg masses, each containing approximately 60 eggs of varying ages (1, 3, and 6 days old). Egg masses were collected from healthy *B. alexandrina* snails maintained in laboratory conditions and laid on foam substrates. The egg masses were continuously exposed to 100 mL of filtrate at LC_o_, LC_10_, LC_25_, LC_50_, and LC_90_ concentrations in Petri dishes until hatching. A control group consisting of egg masses exposed to dechlorinated tap water was similarly maintained (Osman et al. 2013). The egg masses were examined daily under a stereomicroscope, recording the number of viable eggs and successfully hatched embryos (Mostafa and Gawish 2009). Hatchability percentages were calculated by dividing the number of hatched embryos by the total number of eggs at the beginning of the experiment.

### **Toxicity of the*CFS of S. rochei*to*Shistosoma mansoni*larval stages (Miracidia & Cercariae)**

Larvae of *S. mansoni*, including miracidia and cercariae, were procured from the Schistosome Biological Supply Center (SBSC) at the Theodor Bilharz Research Institute (TBRI). The experimental setup included different concentrations of the tested filtrate, corresponding to LC_o_, LC_10_, LC_25_, LC_50_, and LC_90_ values. For each concentration, 10 mL of the filtrate was introduced into a container along with approximately 100 miracidia or cercariae. Control groups consisting of 10 mL of dechlorinated tap water containing the same number of miracidia or cercariae were also prepared. Microscopical observation of larval movement was conducted at various time intervals (15, 30, 45, 60, 120, and 180 min) for both the experimental and control groups. Immobile larvae were considered dead, and their total count was confirmed at the end of the experiment by adding an iodine solution for visualization (Abdel-Motleb et al. 2022).

### Histological study

The histopathological effects of the hermaphrodite and the digestive glands were assessed to observe possible changes that occurred as a result of the exposure to the tested filtrate of *S. rochei* compared to the control snails. *B. alexandrina* (8–10 mm) snails were exposed for 7 days in triplicates (each of 10 snails) for LC_25_ of the tested filtrate. Another group (in triplicates) was the control. The shells of snails were gently crushed between two glass slides and the soft parts of the snails were carefully removed out of the shell. The hermaphrodite and digestive glands of each snail were gently separated and then fixed in Bouin’s solution. The glands were dehydrated using graded ethyl alcohol, cleared in xylene, embedded in paraffin wax, and finally sectioned at 5 μm thickness. Sections were stained with Hematoxyline and Eosin stain (H-E), dehydrated in graded alcohol, cleared in xylene, and mounted in Canada balsam, then microscopically examined. Histological sections were photographed using Olympus bx.41, Japan microscope, photo automated camera (Mansour et al. 2024).

### Chitinase production assay using colloidal chitin-enriched nutrient agar

Nutrient agar media enriched with 0.1% colloidal chitin was utilized to investigate the chitinase production of *S. rochei*. Colloidal chitin was prepared following the method of (Wu et al. 2009). Briefly, 30 g of chitin flakes were suspended in 200 mL of HCl and incubated at room temperature for 2 h on a rotary shaker until fully dissolved. The resulting solution was poured into 1 L of water with continuous stirring to precipitate colloidal chitin. The precipitate was centrifuged at 7000 rpm for 10 min at 4 °C and subsequently washed multiple times with water to adjust the pH to 2.0–3.0. Neutralization was achieved using 1 M NaOH, followed by another round of centrifugation (7000 rpm for 10 min at 4 °C) and washing 2–3 times with sterile water to obtain low-salt colloidal chitin. The final product was dried in an oven at 60 °C for 24 h to produce chitin pills.

*S. rochei* cultures were incubated on the colloidal chitin-enriched nutrient agar for four days at 30 °C. To visualize chitinase activity, the plates were stained with 0.1% Congo red, followed by destaining with 1% NaCl. The appearance of a distinct halo zone around the colonies indicated chitinase synthesis ( Setyahadi et al. 2019).

### Statistical analysis

Results are expressed as means of three different experiments ± standard deviation (SD). Results were statistically analyzed using two way ANOVA and Tukey’s multiple comparisons test. The analysis was performed using GraphPad Prism version 6.04 for Windows, www.graphpad.com.

## Results

### Isolation and screening of actinomycetes antimicrobial activity

**Fig. 1:**
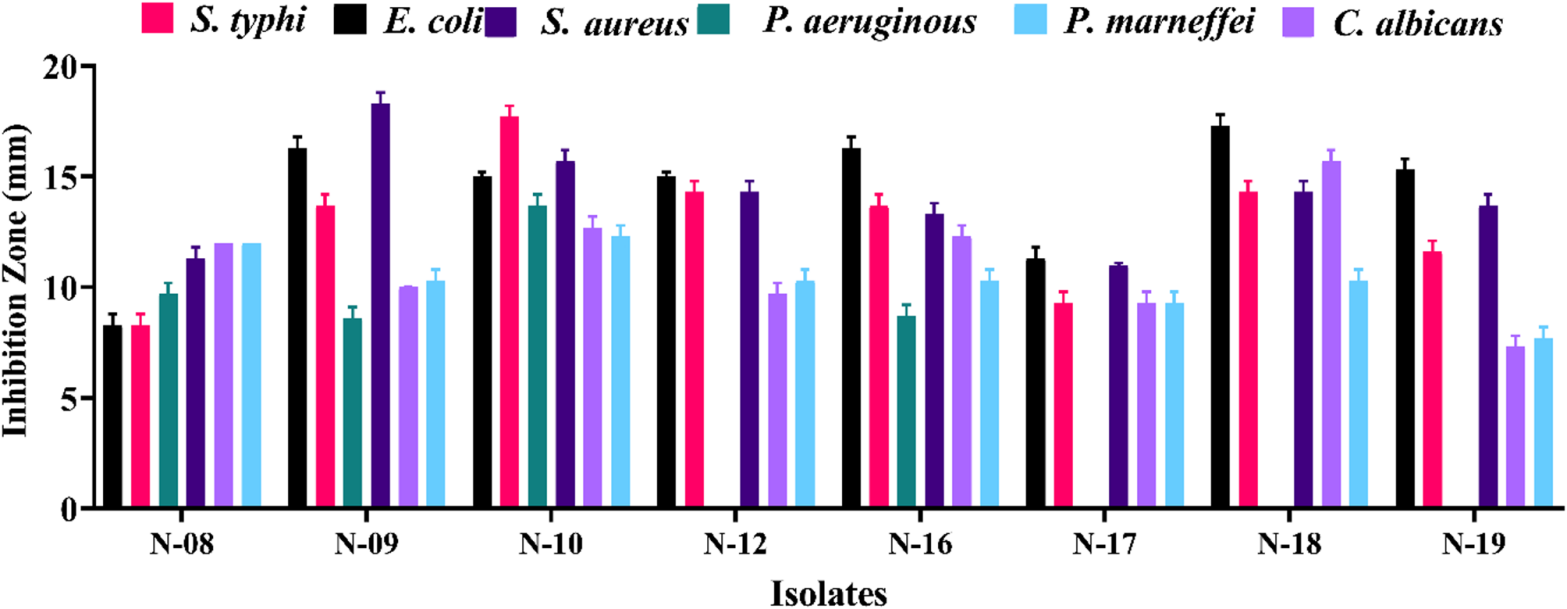
Antimicrobial activity of actinomycetes isolates against pathogenic microorganisms

Nineteen actinomycete isolates from different soil environments in Egypt have antagonistic activity against six pathogenic bacteria, including *E. coli*,* S. typhi*,* P. aeruginosa*,* S. aureus*,* C. albicans*, and *P. marneffei*, as shown in Fig. 1. While some isolates showed no action (data not shown), others, including isolates N-. 08, 09, 10, 12, 16, 18, and 19, demonstrated detectable inhibitory zones. Notably, isolate N- 09 was the most effective against *S. aureus* ) 18.3 ± 0.5 mm), whereas isolate No. 18 displayed the biggest zones of inhibition against *S. typhi* ) 14.3 ± 0.5 mm) and *C. albicans* ) 15.7 ± 0.5 mm). Isolate N-10 was chosen for additional examination out of all of them because of its potent and all-encompassing antimicrobial activity against bacterial and fungal infections. Figure 1 shows detailed measurements of the inhibitory zone.

### Molecular identification of the isolate N-10

**Fig. 2:**
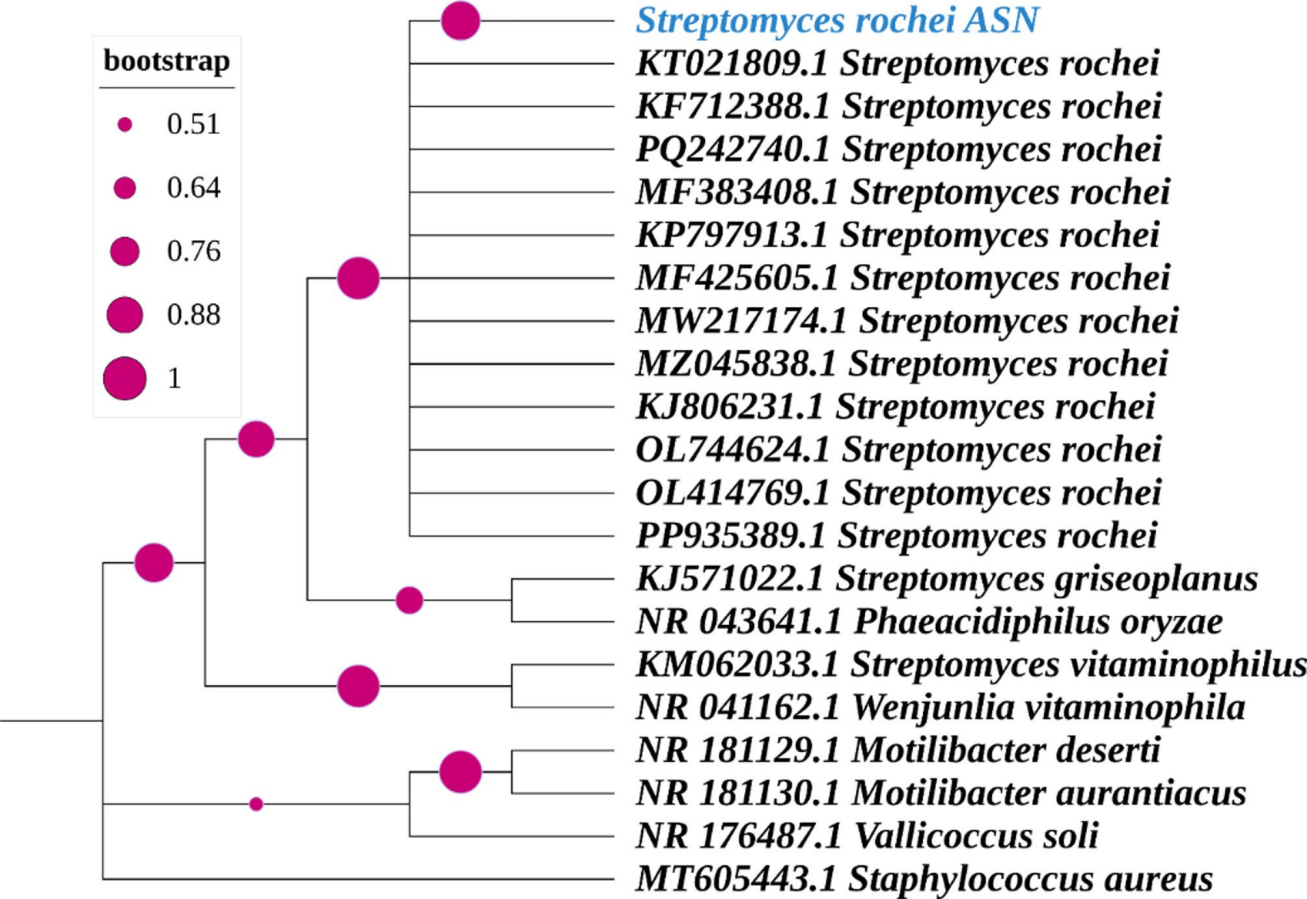
Phylogenetic Tree of *S. rochei ASN* And Related Species Constructed Using 16 S rDNA and Maximum Likelihood Analysis

The phylogenetic analysis represented in Fig. 2 highlights the evolutionary relationship of Isolat N-10 with other related actinomycetes and bacterial species. The tree was constructed using 16 S rDNA sequences and the Maximum Likelihood approach, employing a pairwise distances matrix based on the Jukes-Cantor model. Bootstrap values, displayed next to the branches, represent the proportion of duplicate trees in which the associated taxa clustered together during the bootstrap test (1,000 repetitions), providing statistical confidence for each branching point (Felsenstein 1985). The analysis included 21 nucleotide sequences, allowing less than 5% alignment gaps, missing data, and ambiguous bases at any position (partial deletion option), with all positions having less than 95% site coverage excluded. The final dataset comprised 1122 nucleotide positions. Evolutionary analysis was performed using MEGA11 (Tamura et al. 2021). Notably, *S. rochei* ASN (isolate N-10) was placed within a well-defined clade, indicating a close genetic relationship with other *S. rochei* strains. This analysis confirms the taxonomic identity of *S. rochei* ASN and establishes its evolutionary placement within the genus, reinforcing its classification as a distinct and closely related member of the *Streptomyces* lineage.

### Optimizing antimicrobial activity of *S. rochei* ASN

To improve the production of antimicrobial metabolites, it was crucial to optimize the production medium. This was achieved through a classical experimental approach, where one factor was varied at a time while keeping all other factors at a specific level. By systematically altering and studying individual factors, we aimed to gain a better understanding of the factors influencing antimicrobial metabolite production and develop an optimizing production medium for improved yields (Kennedy and Krouse 1999). As it is known the composition of the growth medium, duration of cultivation, carbon source, nitrogen source, and temperature are among the most critical factors that influence the expression of antibiotic properties in actinomycetes (Waithaka 2022).

**Fig. 3:**
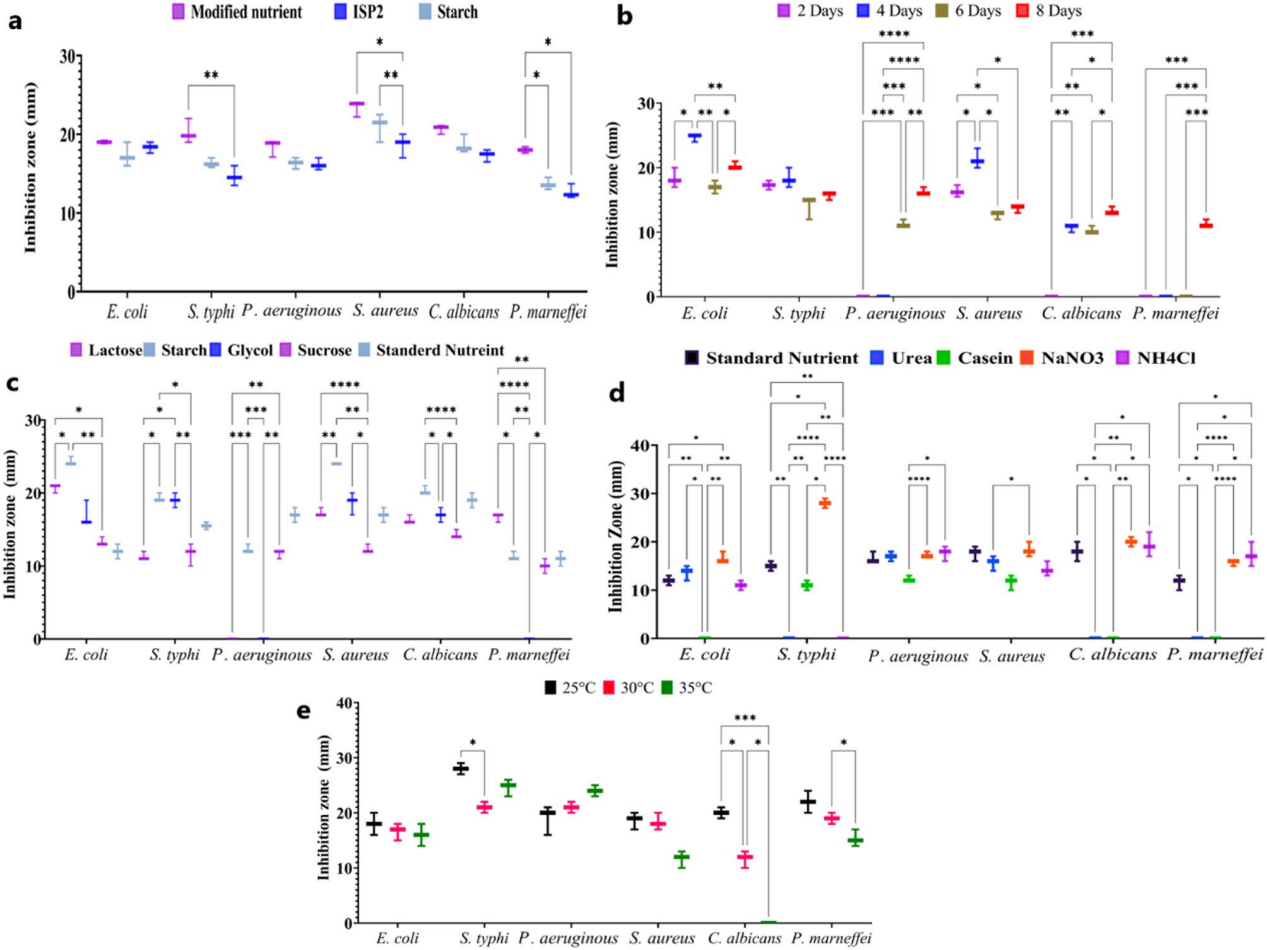
Box-and-whisker plot illustrating the effect of different culture conditions on the antimicrobial activity of the cell-free supernatant of *S. rochei* ASN against different pathogenic microorganisms. The whiskers show the range, while the box represents the interquartile range with the median. **a** Culture media; **b** Incubation periods; **c** Carbon sources; **d** Nitrogen sources; **e** Tempreture. P-value (Two way ANOVA) between all groups < 0.01. Tukey’s multiple comparisons test was conducted, (*) means P-value < 0.05, (**) means 0.05 < P-value ≤ 0.01, (***) means 0.01 < P-value ≤ 0.001. (****) means 0.001 < P-value ≤ 0.0001

The impact of several culture medium on *S. rochei* ASN’s antibacterial activity is depicted in Fig. 3a. The findings highlight the significance of dietary composition in regulating the generation of bioactive metabolites by showing a significant variation in inhibitory zone widths across the tested media. Modified nutritional agar demonstrated the strongest antibacterial efficacy among the assessed media, especially against *S. aureus* )23.4 ± 1.01 mm) and *C. albicans* ) 20.7 ± 0.58 mm). These results demonstrate how *S. rochei* ASN’s manufacture of antimicrobial chemicals can be greatly increased by optimising growth conditions.

The incubation time had an impact on *S. rochei*’s antibacterial activity, as seen in Fig. 3b. After four days of incubation, the isolate showed antagonistic effects and selective activity against specific infections. Interestingly, at this early stage, it did not exhibit any activity against *P. aeruginosa* and *P. marneffei*. Nevertheless, *S. rochei* showed antibiotic action against all six studied pathogens by the eighth day as following *P. aeruginosa* (16.3 ± 0.58 mm), *S. aureus* (13.6 ± 0.58 mm), *C. albicans* (13.3 ± 0.57 mm), *P. marneffei* (11.3 ± 0.57 mm), *E. coli* (20.3 ± 0.57 mm), and *S. typhi* (15.6 ± 0.57 mm), suggesting that prolonged incubation increases the synthesis of bioactive compounds. These findings highlight how crucial incubation duration is to maximising *S. rochei*’s antibacterial activity.

In bacteria, carbon is essential for energy metabolism and the synthesis of secondary metabolites. Lactose, sucrose, starch, and glycol were substituted for glucose in the modified nutritional broth in this investigation in order to assess their effects on *S. rochei*’s antibacterial activity. The most efficient carbon source was starch, which produced much larger inhibition zones against each of the six tested pathogens *E. coli* (24.3 ± 0.58 mm), *S. typhi* (19.3 ± 0.58 mm), *P. aeruginosa* (12.3 ± 0.58 mm), *S. aureus* (24 ± 0.11 mm), *C. albicans* (20.3 ± 0.51), and *P. marneffei* (11.3 ± 0.23 mm), as seen in Fig. 3c. These results imply that the metabolic pathways involved in the creation of bioactive compounds are directly impacted by the availability of carbon. Interestingly, starch significantly increased antibacterial activity, whereas glucose and sucrose had relatively less of an impact.

The impact of several nitrogen sources on *S. rochei*’s antibacterial activity is depicted in Fig. 3d. Sodium nitrate (NaNO₃), which increased antagonistic activity especially *S. typhi* (28 ± 1 mm), *P. aeruginosa* (17.33 ± 0.58 mm), and *S. aureus* (18.33 ± 1.53 mm), was the most effective of the studied sources. While urea showed no inhibition against *S. typhi*,* C. albicans*, and *P. marneffei*, it did support modest action against a small number of pathogens. Among a number of pathogens, ammonium chloride (NH₄Cl) showed moderate to strong activity, particularly against *P. aeruginosa*,* S. aureus*, and *C. albicans*. These findings demonstrate how important choosing the right nitrogen source is to maximising *S. rochei*’s synthesis of antibiotic metabolites. Casein, on the other hand, only had weak activity against a small number of infections and had limited efficacy. As a baseline, standard nutritional medium showed moderate suppression of all examined pathogens, albeit generally less than that of NaNO₃ and NH₄Cl. These results highlight the significance of choosing a nitrogen source since it has a direct impact on the metabolic pathways that lead to the formation of secondary metabolites. NaNO₃ was the most successful nitrogen source evaluated in boosting *S. rochei*’s antibacterial activity, especially against a wide range of pathogenic pathogens.

One important element affecting microbial growth, morphology, and metabolite synthesis is temperature. *S. rochei*’s antimicrobial activity was temperature-dependent, as seen in Fig. 3e, with the best overall inhibition across the majority of tested pathogens occurring at 25 °C. Strong activity was seen at this temperature, especially against *P. marneffei* (22 ± 1.15 mm), *C. albicans* (19.67 ± 0.57 mm), and *S. typhi* (28 ± 0 mm). With the exception of *P. aeruginosa* (18.67 ± 2.31 mm), whose inhibition rose with temperature, antimicrobial activity generally decreased as temperatures rose. These results imply that 25 °C is the ideal temperature for *S. rochei* to produce the most bioactive compounds, while particular reactions may differ based on the target organism.

### Impact of different concentrations of *S. rochei* cell-free supernatant on pathogenic microorganisms growth

The effects of different doses of *S. rochei* cell-free supernatant (CFS) (20%, 60%, and 80%) on the dry weight and inhibition percentage of several bacterial and fungal pathogens are shown in Table 1. Higher CFS concentrations result in larger decreases in microbial biomass and enhanced inhibition, according to the results, which clearly show a dose-dependent response. With significant dry weight decreases and high inhibitory percentages at 80% concentration, *S. aureus*,* P. marneffei*, and *C. albicans* demonstrated the highest sensitivity to the CFS among the studied species. According to these results, the metabolites of *S. rochei* are very effective against fungi and Gram-positive bacteria, and their bioactivity increases with concentration.

**Table 1:** Effect of different concentration of Cell-Free supernatant (CFS) of *Streptomyces rochei* ASN on biomass reduction of pathogenic microbes

		Dry weight (mg/ml)	Inhibition %				
		CFS (20%)	CFS (60%)	CFS (80%)	CFS (20%)	CFS (60%)	CFS (80%)
*E. Coli*	Control	3.31			–		
	Treated	2.17	1.64	0.58	34.44	50.45	82.48
*S. typhi*	Control	2.99			–		
	Treated	1.61	1.27	0.60	46.15	57.53	79.93
*P. aeruginosa*	Control	3.52			–		
	Treated	1.92	1.57	0.88	45.45	55.40	75.00
*S. aureus*	Control	4.15			–		
	Treated	1.52	1.20	0.58	63.37	71.08	86.02
*C. albicans*	Control	13.38			–		
	Treated	5.73	4.50	2.11	57.17	66.37	84.23
*P. marneffei*	Control	13.03			–		
	Treated	4.70	3.80	2.01	63.93	70.84	84.57

### Scanning electron microscope (SEM) analysis of morphological alterations in pathogenic microorganisms treated with *S. rochei* cell-free supernatant

**Fig. 4:**
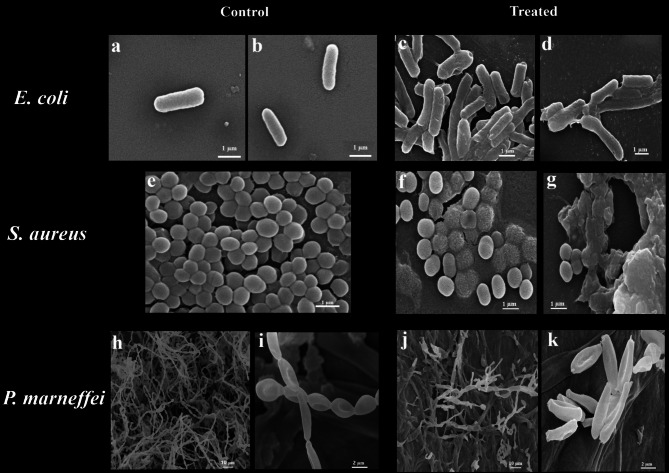
Scanning electron microscopy (SEM) images demonstrating the antimicrobial activity of *S. rochei* against pathogenic microorganismsa: **a**,**b** Control cells of *E. coli*. **c**,**d** Treated cells of *E. coli*. **e** Control cells of *S. aureus*. **f**,**g** Treated cells of *S. aureus* (h, i) Control cells of *P. marneffei*. (j, k) Treated cells of *P. marneffei*

Scanning electron microscopy (SEM) was employed to investigate the morphological alterations in *S. aureus*, *E. coli*, and *P. marneffei* following treatment with 20% cell-free supernatant (CFS) of *S. rochei* Fig. 4. Significant morphological changes were observed in all treated pathogens. In *E. coli*, SEM images revealed extensive cellular damage, characterized by visible disintegration, irregular cell shapes, and cell adhesion, potentially indicative of stress-induced aggregation (Fig. 4a-d). Similarly, treated *S. aureus* cells exhibited abnormal morphology and separation compared to the dense clusters observed in the control group, suggesting potential disruption of biofilm structures or interference with cell-cell adhesion mechanisms (Fig. 4e-g).

In the case of *P. marneffei*, SEM analysis revealed varying degrees of hyphal degradation, including irregular, fractured, and thinner hyphae compared to the control, suggesting potential targeting of the cell wall or membrane (Fig. 4h-k).

### Molluscicidal activity *CFS of S. rochei*

The molluscicidal activity of *S. rochei* filtrate against *B. alexandrina* snails after 24 h of exposure followed by 24 h recovery period under laboratory conditions has been shown in Table 2. The obtained results indicated that the LC_50_ and LC_90_ values for the tested filtrate were 26.85& 40.95ppm, respectively. The sub-lethal concentrations LC_o_, LC_10_, and LC_25_ were 2.86, 12.74 & 19.42 ppm, respectively.

**Table 2:** Molluscicidal activity of *S. rochei* cell-free supernatant (CFS)against adult *B. alexandrina* snails

*S. rochei* CFS	LC_50_ (ppm)	LC_90_ (ppm)	Sublethal concentrations (ppm)		
			LC_o_	LC_10_	LC_25_
	26.85	40.95	2.68	12.74	19.42

### **Effect of CFS*of S. rochei*on hatchability of*B. alexandrina*eggs**,** miracidicidal and cercaricidal activity**

**Fig. 5:**
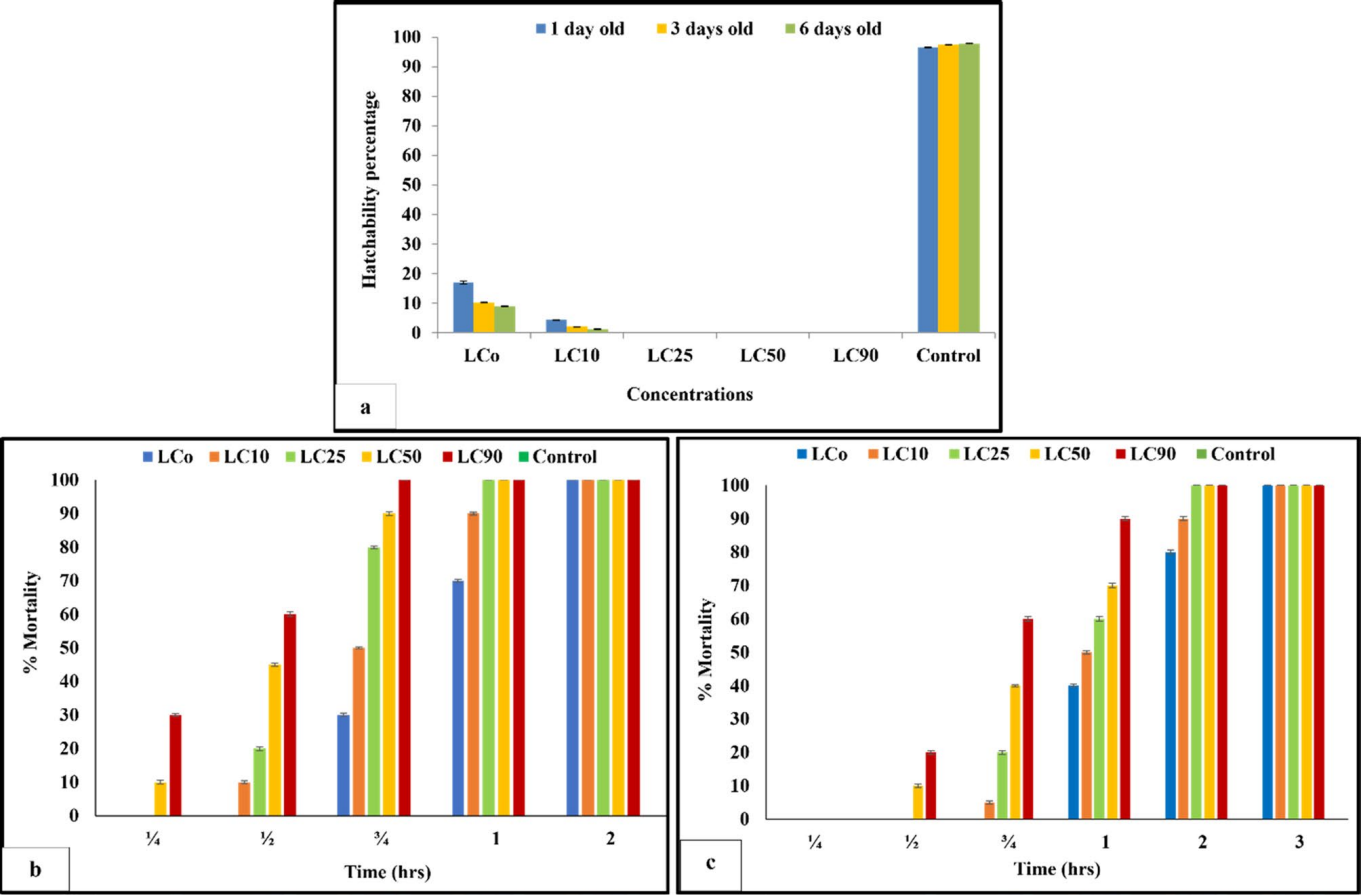
Effect of CFS *of S. rochei* on hatchability of *B. alexandrina* eggs (**a**), miracidicidal (**b**) and cercaricidal (**c**) activity

For the hatchability test, the snail’s eggs at one, three, and six days’ ages were exposed to LCo, LC_10_, LC_25_, LC_50_, and LC_90_ of the tested filtrate Fig. 5a. The data revealed that the eggs of different ages didn’t hatch in the examined solution, except eggs at one, three, and six days ages exposed to LCo and LC_10_ of the tested filtrate, where the hatchability percents were (16.95, 10.34 & 8.02%) and (4.33, 2.05 & 1.28%) compared to 96.45, 97.36 and 97.89% for control groups, respectively. Meanwhile, eggs of six days of age were more susceptible than those of one and three days of age. The hatchability percent of the different groups of exposed eggs was significantly decreased by their age and filtrate concentrations.

Regarding the miracidial and cercaricidal activity, the data revealed that *S. rochei* cell-free supernatant (CFS) excerted high mortalities of miracidia Fig. 5b and cercariae Fig. 5c. The filtrate exerted a miracidial effect after 1/4 hour of their exposure to LC_50_ and LC_90_ ppm of the experimental extract. Also, 100% of *S. mansoni* miracidia were killed after 1 h of exposure to LC_25_, LC_50_, and LC_90_ ppm of the tested extract. While the mortality rate of cercariae reached 100% after 2 h of exposure to the same concentrations. The tested filtrate showed cercaricidial effect after 1/2 hrs of exposure to LC_50_ and LC_90_ ppm. In general, the miracidia are more sensitive to the toxic action of the tested agent than cercariae and the mortality percent of miracidia and cercariae is directly proportional to the time and the tested concentrations.

### Histological study

The histopathological alterations were studied in both hermaphrodite and digestive glands of *B. alexandrina* snails at 7 days post-exposure to LC_25_ (19.42 ppm) of *S. rochei* filtrate.

A. Hermaphrodite gland

**Fig. 6:**
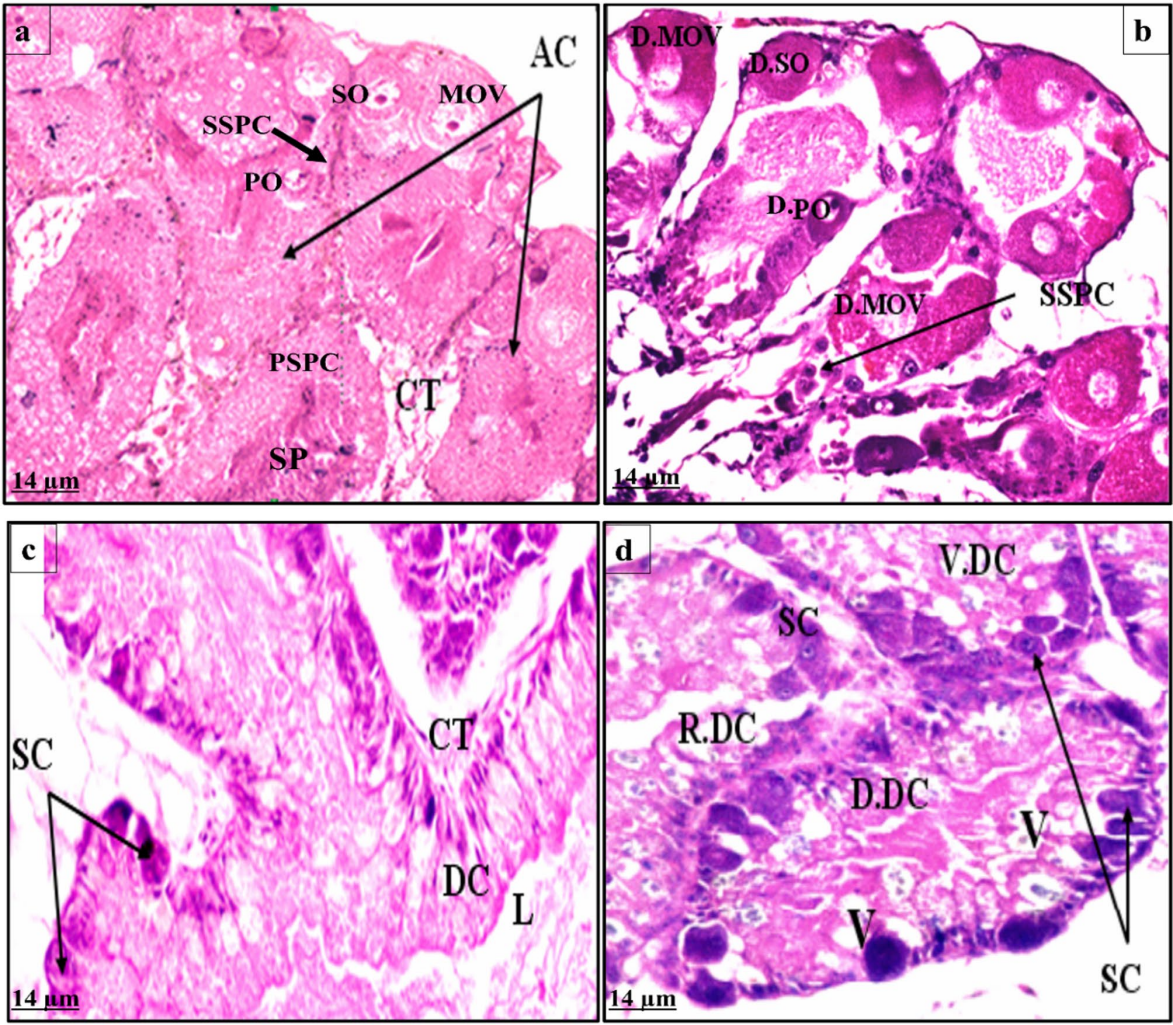
**a** Phototmicrograph of section through control hermaphrodite gland of *B. alexandrina* snails showing simple branched acini (AC), connective tissue (CT), stages of spermatogensis (primary spermatocytes (PSPC), secondary spermatocytes (SSPC) and sperms (SP)), stages of oogenesis (primary oocytes (PO), secondary oocytes (SO) and mature ova (MOV)). **b** Photomicrograph of section through hermaphrodite gland of *B. alexandrina* snails treated with LC_25_ of *S. rochei* cell-free supernatant (CFS)showing degeneration of primary oocytes (D.PO), secondary oocytes (D.SO) and mature ova (D.MOV). Secondary spermatocytes (SPSC) become dispersed outside acini. **c** Photomicrograph of section through control digestive gland of *B. alexandrina* snails showing digestive cells (DC), secretory (SC), the lumen (L) and connective tissue (CT). **d** Photomicrograph of section through digestive gland of *B. alexandrina* snails treated with LC_25_ of *Streptomyces rochei* cell-free supernatant (CFS) showing vacuoles (V). Rupture of some digestive cells (R.DC), some became vacuolated (V.DC) and others are degenerated (D.DC). Secrtory cells suffered from disfiguring of their shape, became denser in color and some of them missed their nuclei (SC) (H & E stain)

The control hermaphrodite gland of *B. alexandrina* snails is composed of simple branched acini (AC) which are connected to each other by a thin layer of connective tissue (CT). The acini contain different stages of spermatogensis; primary spermatocytes (PSPC), secondary spermatocytes (SSPC), and sperms (SP). The stages of oogenesis are also represented in most of the acini as primary oocytes (PO), secondary oocytes (SO), and mature ova (MO) Fig. 6a. On the other hand the treatment of *B. alexandrina* snails with LC_25_ of the tested filtrate for 7 days lead to disturbance in the normal shape of acini and degeneration of the membranes of acini. Degeneration in primary oocytes (D.PO) secondary oocytes (D.SO) and mature ova (D.MOV) as they lost their nulclei, identical shape and also their yolk layers are ruptured. Also, the male gonadal cells are not safe from the effect of Score testing agent where spermatocytes (secondary spermatocytes SPSC) become dispersed outside acini and most of the spermatogenic stages and sperms disappeared Fig. 6b.

B. Digestive gland

The control digestive gland of *B. alexandrina* snails consists of a number of tubules which are connected by connective tissue. Each tubule consists of two main cell types; the digestive and secretory cells. The digestive cells (DC) are the most numerous, they are columnar with round apices, and the cytoplasm with numerous vesicles of different sizes, their nuclei are oval and lie in the basal region. In between the digestive cells, the secretory (SC) ones are distributed in the acinus in smaller numbers. They are pyramidal in shape, their cytoplasm is highly basophilic and contain a large number of chromatin granules, note the lumen (L) between acini and connective tissue (CT) Fig. 6c. While the treatment of snails of the tested filtrate for 7 days exerted disturbance of normal articture of digestive tubules and intensive damage as most of the connective tissue was broken down so the vacuoles became distinctive (V). Rupture of some digestive cells (R.DC), some became vacuolated (V.DC) with ruptured tips, and others lost their identical shape resulting from degeneration of their cell membrane (D.DC). Secrtory cells suffered from disfiguring of their shape, became denser in color and some of them missed their nuclei (SC) Fig. 6d.

### Chitinase production assay using colloidal Chitin agar

Since the pathogens are greatly affect by chitinase, the isolation of endophytic chitinolytic actinomycetes is essential to this investigation. The ability of actinomycetes on the production of chitin was tested on Colloidal Chitin Agar. The diameter of the clear zone formed due to the production of chitinase was 23 mm (Fig. 1, supplementary material).

## Discussion

Our findings indicate that the antimicrobial efficacy of *S. rochei* ASN is markedly influenced by the cultivation conditions. In terms of medium composition, our results align with those of Vijayakumar et al. (2012), who demonstrated enhanced antifungal activity in a modified nutritional medium. This observation corroborates the work of Sebak et al. (2021), who reported that ISP4 medium was most effective in supporting the antimicrobial potential of *Streptomyces* sp. MS10 against a broad spectrum of pathogens. Conversely, Salem et al. (2023) found that although nutritional agar exhibited only moderate antibacterial effects, media such as Bennett’s and ISP2 facilitated sustained microbial growth. Similarly, Al-Ghazali et al. (2017) observed SG medium to be optimal for targeting *E. coli* and *P. aeruginosa*, whereas Djinni et al. (2023) identified Czapeck medium as superior for enhancing *S. rochei* CMB47 activity against MRSA. These contrasting outcomes collectively underscore the strain- and pathogen-specific variability in medium efficacy, highlighting the importance of customized screening protocols for optimizing antimicrobial production.

Regarding incubation time, our data revealed that four-day cultures were effective against certain pathogens, while extending the incubation to eight days broadened the spectrum of inhibition across all tested microorganisms. These findings resonate with Ahmad et al. (2017), who reported an incremental increase in antimicrobial inhibition from day three, reaching a peak by day eleven. Similarly, Reddy et al. (2011) documented the initial signs of antimicrobial activity by *S. rochei* as early as days two to three. This early onset implies that the biosynthesis of bioactive secondary metabolites is initiated relatively early in the growth cycle. Djinni et al. (2023) also reported early and peak antimicrobial activity around days two and three, suggesting a tight linkage between metabolite production and the transitional phases of microbial growth.

Carbon source selection emerged as another critical factor influencing antimicrobial metabolite production. In agreement with Reddy et al. (2011), our findings showed that complex carbohydrates such as starch significantly enhanced secondary metabolism. This effect may be attributed to the high amylolytic activity of *S. rochei*, as documented by Al-Agamy et al. (2021). Comparable results were reported by Rakesh et al. (2013) and Osman et al. (2011), who also noted that starch was optimal for their respective strains. Nonetheless, Mariadhas et al. (2014) identified glucose and fructose as superior for certain isolates, reinforcing the notion of strain-specific preferences in carbon metabolism.

Nitrogen source variation likewise had a pronounced effect. Our observations support prior studies by Ababutain et al. (2013) and Mariadhas et al. (2014), which demonstrated a preference for inorganic nitrogen forms such as sodium nitrate in promoting both microbial growth and antimicrobial activity. Furthermore, Elabbasy et al. (2021) identified potassium nitrate (KNO₃) as the most effective antifungal nitrogen source. Complementarily, Sarika et al. (2021) reported enhanced activity in *Streptomyces* BHPL-KSKU5 when supplemented with NaNO₃ and asparagine. These collective findings reaffirm that both the form and concentration of nitrogen are pivotal in modulating bioactive metabolite synthesis, with our study identifying an optimal pH of 7.0 for maximal activity.

Temperature optimization confirmed the thermotolerant nature of *S. rochei* ASN, with peak antimicrobial performance recorded at 25 °C. This is in line with the findings of Tenebro et al. (2021), although other investigations have reported optimal activity at slightly elevated temperatures for different *Streptomyces* strains (Augustine et al. 2005; Ripa et al. 2009; Reddy et al. 2011; Mariadhas et al. 2014). This variation suggests a degree of physiological and metabolic adaptability linked to each strain’s ecological origin.

Across all test species, the cell-free supernatant (CFS) derived from *S. rochei* ASN exhibited pronounced antimicrobial properties, with fungi generally showing greater susceptibility than bacteria. These results are consistent with earlier reports by El-Abyad et al. (1993), which highlighted the broad-spectrum antifungal activity of *Streptomyces* filtrates. Similar antimicrobial efficacy was observed by Girma and Aemiro (2022**)** and Verma et al. (2024), who reported strong antibacterial activity from *Streptomyces* isolates, including resistance-breaking strains such as MRSA and VRE. Girma and Aemiro (2022**)** also documented significant inhibitory effects of soil-derived *Streptomyces* on foodborne pathogens like *Listeria monocytogenes*, *S. typhi*, and *E. coli*, affirming their potential in antimicrobial drug development. Verma et al. (2024) further confirmed the robust activity of *Streptomyces levis* CFS, with inhibition zones reaching up to 28 mm against various clinical pathogens. In our investigation, CFS at 60% concentration led to over 50% suppression of all tested pathogens, underscoring the high antibacterial potential of *S. rochei* ASN.

With regard to antibiofilm capabilities, our CFS markedly impaired biofilm formation, consistent with findings by Dar and Ahmad (2024**)**, who demonstrated similar activity from *Streptomyces rameus*. Scanning electron microscopy (SEM) revealed clear structural disruption in bacterial biofilms, echoing mechanisms described by Raissa et al. (2020). Additionally, evidence of fungal membrane perturbation mirrored the hyphal deformation and spore reduction reported by Meena et al. (2022) and Evangelista-Martínez et al. (2020) following treatment with *Streptomyces* metabolites.

The molluscicidal activity of *S. rochei* ASN against *Biomphalaria alexandrina* was substantiated by LC_50_ and LC_90_ values of 26.85 and 40.95 ppm, respectively. The concentrations (LC_25_, LC_50_, LC_90_) notably stopped egg hatchability. Biological control using microbial metabolites has shown promising ovicidal and molluscicidal effects against *B. alexandrina*. These compounds interfere with the snails’ hormone levels, liver enzyme activity, and hemolymph protein patterns, disrupting vital biological functions and reducing survival and reproduction (Mekawey et al. 2022). our outcomes are in agreement with those observed for fungal and *Bacillus*-derived molluscicides (Osman et al. 2013; Saleh et al. 2022) and are corroborated by reports from El-Bolkiny et al. (2000), Abouel-Hassan et al. (2000), and Al-Mathal and Fouad (2006), who also documented adverse impacts on snail embryogenesis. The miracidicidal and cercaricidal activities of the tested filtrate revealed miracidial mortalities greater than that of cercariae after the same time intervals and the mortality percents were directly proportional to the time and the concentrations tested, suggestive of miracidial sensitivty towards the toxic action of the tested agent than cercariae, as described by Tantawy (2006). Similar observations were observed by Osman et al. (2013), Abou-Elnour et al. (2015) and Abdel-Motleb et al. (2022).

Histopathological assessments revealed significant tissue alterations in snail organs exposed to LC₂₅ of *S. rochei* ASN CFS. The hermaphrodite gland showed disrupted acinar structures, degenerative ova, and impaired gametogenesis, suggestive of endocrine interference, as described by Chaffai and Ismail (2010). Comparable damage was also observed by Mossalem et al. (2013), Abd El-Halim Saad et al. (2016), and Saleh et al. (2022). The digestive gland exhibited degradation in connective tissue and secretory and digestive cell integrity, in concordance with hepatotoxic effects reported by Lajtner et al. (1996) and Abdel-Wareth et al. (2019).

Additionally, *S. rochei* ASN demonstrated robust chitinase activity, as evidenced by the formation of clear zones on colloidal chitin agar. This supports the established role of chitinase in fungal cell wall degradation for nutrient access (Prasad et al. 2021) and nematode control (Fan et al. 2023). Similar antifungal capabilities have been attributed to *Streptomyces* strains in the works of Rodrigues et al. (2018), Liu et al. (2019), and Rajendran et al. (2023). Our results align with previous reports from Musa et al. (2020) and El-Akshar et al. (2024), highlighting chitinase as a key effector in *S. rochei*’s antifungal activity. Although chitinase production is a well-recognized contributor to antifungal efficacy, recent studies suggest the involvement of additional bioactive compounds. For instance, Zhou et al. (2024) reported that *S. rochei* produces antibiotics such as borrelidin with broad-spectrum activity. Furthermore, bioactives with nematicidal, antifungal, and insecticidal properties have been identified in this species (Salaskar et al. 2024; Mani et al. 2025), indicating a complex secondary metabolite profile.

Based on these findings, we hypothesize that the observed molluscicidal and antimicrobial activities result from a synergistic interplay between chitinase and other secondary metabolites, potentially including proteolytic enzymes or membrane-disrupting agents. Ongoing chemical and biochemical investigations.

## Conclusion

This study underscores the remarkable potential of *S. rochei* as a powerful dual-action agent with significant antimicrobial and molluscicidal activities, offering a novel and eco-friendly approach to integrated disease management. Among the 19 isolated actinomycetes, *S. rochei* exhibited broad-spectrum antimicrobial activity against Gram-positive bacteria, Gram-negative bacteria, and fungal pathogens, achieving optimal performance under specifically tailored culture conditions. These included the use of starch as the carbon source, sodium nitrate as the nitrogen source, and an incubation temperature of 25 °C in modified nutrient agar. Its molluscicidal efficacy against *B. alexandrina* was profound, with LC_50_ and LC_90_ values calculated at 26.85 and 40.95 ppm, respectively, and notable toxicity against *S. mansoni* miracidia and cercariae, with mortality rates increasing over time and concentration. The production of chitinase enzyme by *S. rochei* was a key factor in its antifungal and molluscicidal effects. Scanning Electron Microscopy revealed significant morphological disruptions in treated pathogens, including cell wall damage, cytoplasmic leakage, and structural disintegration, confirming its potent bactericidal and fungicidal mechanisms. Histological analyses of the CFS against *B. alexandrina* further highlighted severe tissue degeneration in the hermaphrodite and digestive glands of snails exposed to sublethal concentrations of *S. rochei* filtrate, including vacuolation, cell membrane damage, and the destruction of reproductive and digestive cells. These findings suggest *S. rochei* as a promising eco-friendly candidate for developing sustainable applications in agriculture and pharmaceuticals, providing an effective alternative to chemical molluscicides and paving the way for further research into its molecular mechanisms and scalability for commercial use.

### Limitations of the study

This study presents promising antimicrobial and molluscicidal properties of *S. rochei* ASN; however, the following limitations should be noted. The actinomycete isolates were collected from limited geographic locations, potentially limiting strain diversity and overlooking other bioactive candidates. Identification of the selected isolate was based solely on 16 S rRNA sequencing; whole-genome analysis was not performed, restricting insight into biosynthetic gene clusters responsible for metabolite production.

Optimization experiments were confined to controlled laboratory conditions, thus, the findings may not fully translate to field conditions. Moreover, the bioactivity assays relied on crude cell-free supernatant (CFS), and the active compounds were not chemically identified or characterized, preventing precise understanding of their structure or mechanism of action.

The molluscicidal assays were conducted only on *Biomphalaria alexandrina* and *Schistosoma mansoni* larvae under laboratory settings without field validation or broader ecological impact assessments.

Further investigations involving metabolite profiling, in vivo testing, and ecological safety evaluations are required to support the practical application of *S. rochei* ASN in antimicrobial and vector control strategies.

## Supplementary Information

Below is the link to the electronic supplementary material.

Supplementary Material 1

## Data Availability

Data is provided within the manuscript or supplementary information files.
