# Correction: Antimicrobial and molluscicidal activities of Egyptian soil-derived *Streptomyces rochei*

**DOI:** 10.1186/s13568-025-01959-6

**Published:** 2025-09-26

**Authors:** Nora Elfeky, Aya Abd Elsalam, Sabha El-sabbagh, Asmaa Abdel-Motleb

**Affiliations:** 1 Botany and Microbiology Department, Faculty of Science, Menoufia University, Menoufia, Egypt; 2 Environmental Research Department, Theodor Bilharz Research Institute, Giza, Egypt

**Correction to: AMB Express (2025) 15:125**

10.1186/s13568-025-01927-0

In the original publication of this article (Elfeky et al. 2025), the author’s name Asmaa Abdel-Motleb was incorrectly written as Asmaa Abd El-Motleb. The original article has been corrected.
