# Supplementary figures and images for: Antimicrobial and molluscicidal activities of Egyptian soil-derived Streptomyces rochei
Source: AMB Express. 2025 Aug 22;15:125. doi: 10.1186/s13568-025-01927-0 (PMC12373970; doi:10.1186/s13568-025-01927-0)

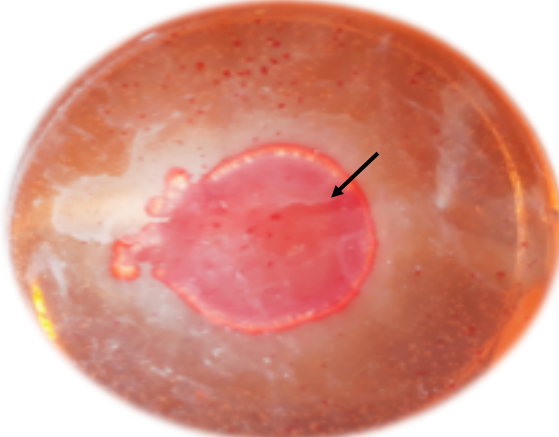


**FIG (1): the presence of clear zone around *S.rochei* on cholidal chitin agar after the destaining.**

Supplement: Supplementary file 1 — Supplementary Material 1 [file 13568_2025_1927_MOESM1_ESM.docx]
